# Glycated albumin and the risk of micro- and macrovascular complications in subjects with Type 1 Diabetes

**DOI:** 10.1186/s12933-015-0219-y

**Published:** 2015-05-15

**Authors:** Hye-jin Yoon, Yong-ho Lee, So Ra Kim, Tyler Hyungtaek Rim, Eun Young Lee, Eun Seok Kang, Bong-Soo Cha, Hyun Chul Lee, Byung-Wan Lee

**Affiliations:** Department of Internal Medicine, Yonsei University College of Medicine, Seoul, South Korea; Severance Hospital, Seoul, Korea, 120-752; Institute of Vision Research, Department of Ophthalmology, Yonsei University College of Medicine, Seoul, Korea, 120-752

**Keywords:** Type 1 diabetes, Glycated albumin, Diabetic nephropathy, Carotid artery atherosclerosis

## Abstract

**Background:**

We investigated the relationship between the glycemic indices glycated albumin (GA) and glycated hemoglobin (HbA_1c_) and the progression of diabetic vascular complications [diabetic nephropathy (DN) and carotid artery atherosclerosis (CAA)] in subjects with type 1 diabetes (T1D).

**Methods:**

A total of 154 participants with a median follow-up of 2.8 years were enrolled in this retrospective longitudinal study. We recruited T1D subjects who had regularly measured urine albumin-creatinine ratios and estimated glomerular filtration rates, as well as tested HbA_1c_ and GA levels consecutively every 3 or 6 months. A subgroup of 54 subjects was measured repeated carotid intima-media thickness (IMT).

**Results:**

We classified subjects into the DN progression (Group I; n = 30) with either deteriorated stages of chronic kidney disease (n = 18) or albuminuria progression (n = 17), and the non-progression (Group II; n = 124). In multiple logistic regression analyses, baseline albuminuria (odds ratio [OR] = 2.64, 95 % confidence interval [CI] = 1.03–6.74), mean GA levels (OR = 2.03, 95 % CI = 1.27–3.26) were significantly associated with progression of DN. However, there was no association with mean HbA_1c_ (OR = 0.98, 95 % CI = 0.62–1.54). In a subgroup analysis for follow-up measurements of carotid IMT, age was independently associated with the presence of plaque and the mean IMT. However glycemic indices were not significantly associated with CAA.

**Conclusions:**

Mean GA levels were more closely associated with DN progression than mean HbA_1c_ in subjects with T1D. However, they were not associated with the CAA.

**Electronic supplementary material:**

The online version of this article (doi:10.1186/s12933-015-0219-y) contains supplementary material, which is available to authorized users.

## Introduction

Current guidelines for glucose monitoring recommend self-monitoring of blood glucose (SMBG) and glycated hemoglobin (HbA_1c_) to accurately assess glycemic status and prevent diabetic complications [1, 2]. Treatment recommendations for type 1 diabetes (T1D) are largely based on the results of the Diabetes Control and Complications Trial (DCCT) and the follow-up of the DCCT cohort for an additional 8 or 16 years in the Epidemiology of Diabetes Interventions and Complications (EDIC) study [3, 4]. These trials showed that the intensive treatment T1D group had lower HbA_1c_ levels, which reduced the risk of microalbuminuria and decreases in the glomerular filtration rate (GFR), compared to the conventional treatment group [2, 3, 5, 6]. Although HbA_1c_ is considered a gold standard measurement for glucose monitoring in patients with diabetes, there is growing evidence regarding the clinical relevance of intermediate-term glycemic index of glycated albumin (GA) for the diagnosis, evaluation for glucose status, and prediction of diabetic complications, particularly in patients with type 2 diabetes (T2D) [7–10]. Recently, Nathan et al. reported the clinical relevance of various glucose biomarkers of short-, intermediate-, and long-term glycemia on micro- and macrovascular complications in subjects with T1D. These authors suggested that HbA_1c_ and GA predicted retinopathy and nephropathy to a greater extent than mean blood glucose values, and the combination of both measurements strengthened the association with retinopathy but not with nephropathy [11]. Based on the results of the DCCT/EDIC study [11], we aimed to determine which glycemic index is more closely associated with the progression of diabetic nephropathy (DN), a microvascular complication, and carotid artery atherosclerosis (CAA), a macrovascular complication, in Korean subjects with T1D.

## Subjects, materials and methods

### Subjects and research design

In this retrospective longitudinal study, we recruited subjects with T1D [12] who were registered in the Severance Hospital Diabetes Registry between August 2009 and December 2013. We used the following inclusion criteria: 1) patients who regularly measured both their spot urine albumin-creatinine ratio (ACR) and kidney function using estimated GFR; and 2) patients who tested HbA_1c_ and GA levels consecutively every 3 or 6 months. The exclusion criteria for this study were patients with non-diabetic kidney disease, end-stage renal disease (ESRD) on dialysis, severe liver disease, pancreatic cancer, hyper or hypothyroidism or acute infectious disease. We classified subjects into two groups: the DN progression group (Group I) and the DN non-progression group (Group II). We defined Group I subjects with deteriorated renal function based on estimated GFR or the advent of albuminuria greater than 30μg/ml or the progression in albuminuria from microalbuminuria [30-300μg/mg ACR] to macroalbuminuria (>300μg/mg, ACR). For deteriorated renal function, we adopted six clinically-relevant Chronic kidney disease (CKD) categories (≥90, 60–89, 45–59, 30–44, 15–29, or <15 mL/min/1.73m^2^) that were assessed using the chronic kidney disease epidemiology collaboration formula (CKD-EPI) [13]. We also defined impaired renal function as decreased CKD stage. For albuminuria, we applied the conventional definition of microalbuminuria as 30μg/mg ≤ urinary ACR ≤ 300μg/mg and macroalbuminuria as ACR >300μg/mg. In logistic regression analyses, we adhered to the previous definition of microalbuminuria as urinary ACR more than 40μg/mg [11]. We conducted additional analyses on the progression of CAA to patients who were repeatedly examined for carotid intima-media thickness (IMT). These subjects were classified into the active CAA and inactive CAA groups. The active CAA group was defined as patients with newly developed carotid artery plaque(s) or persistent ones, whereas inactive CAA group included patients with the absence of carotid IMT plaques or regressed plaques. The Presence of retinopathy was confirmed by an ophthalmologist based on funduscopic findings. The Ethics Committee of the Yonsei University College of Medicine approved this study protocol (2013-0917-001).

### Laboratory measurements

Blood samples were collected from patients after overnight fasting. Serum GA levels were determined using an enzymatic method and an albumin-specific proteinase (ketoamine oxidase), albumin assay reagent (LUCICA GA-L, Asahi Kasei Pharma Co., Tokyo, Japan), and a Hitachi 7699 P module auto-analyzer (Hitachi Instruments Service, Tokyo, Japan) [14]. The coefficient of variation (CV) was 1.43 %. HbA_1c_ was measured using high-performance liquid chromatography (HPLC) and a Variant II Turbo (Bio-Rad Laboratories, Hercules, CA). The reference intervals of HbA_1c_ were between 4.0 and 6.0 %, whereas those of GA were between 11.0 and 16.0 %. The averages of HbA_1c_ (mean HbA_1c_), GA (mean GA) were determined by taking the sum of every measured value in each individual divided by the number of values obtained throughout the study period.

### Measurements of carotid intima-media thickness

Measurements of IMT in both carotid arteries were described in detail previously [15]. Common carotid arterial ultrasound examinations were conducted by two specialized technicians using an Aloka ProSound ALPHA 10 (HITACHI, Tokyo, Japan) with a 13MHz linear probe. IMT was defined as the distance between the media-adventitia interface and the lumen-intima interface. Average IMT was the mean value of computer-based points in the region, and maximal IMT was the IMT value at the maximal point of the region. Plaques were defined according to the Mannheim consensus [16], in which a plaque is diagnosed when the vessel wall thickness was >1.5mm or when the vessel wall appeared to be at least 0.5mm, or 50 % thicker, than the surrounding wall.

### Statistical analyses

All statistical analyses were performed using PASW statistics software (version 20.0; SPSS Inc., Chicago, IL). Continuous variables are described as the mean ± standard deviation (SD) or the median (interquartile range) based on results from Kolmogorov-Smirnov tests. Categorical data are expressed as the number (n) with percentages. Statistical comparisons between groups with and without DN progression were performed using Mann-Whitney U tests or χ^2^ tests, which are non-parametric statistical methods. Multivariate logistic regression analyses were used to estimate multiple correlations between DN progression and clinical and laboratory risk factors. We used receiver operating characteristic (ROC) curve analyses, estimating the area under the curve (AUC) with 95 % confidence intervals (CI), to compare GA levels and HbA_1c_. P values <0.05 were considered significant. In subgroup analyses, we determined the variables that were associated with carotid artery plaques or atherosclerosis progression using multiple linear regression analyses. Logistic regression power calculation was carried out using PASS software version 13.0.10 (NCSS statistical Software, Kaysville, UT).

## Results

### Characteristics of the Study Participants

A total of 154 participants (69 men and 85 women; mean age, 46 ± 15 years) with a median follow-up period of 2.8 years were enrolled in this study. The numbers of subjects that progressed from microalbuminuria to macroalbuminuria and exhibited deterioration of CKD stage were 18 (11.7 %) and 17 (11 %), respectively. Based on these results, we classified subjects into the progressed DN group (Group I; n = 30) and the non-progressed DN group (Group II; n = 124). Table 1 shows the demographic and laboratory characteristics of the subjects. Age, gender, and body mass indices (BMI) were similar between the two groups. The prevalence of hypertension and the use of anti-hypertensive medications did not significantly differ between groups. Group I had a significantly longer diabetic duration [12.0 (10.0–18.0) vs. 8.0 (3.0–15.0) years, P = 0.004] and higher proportion of retinopathy (70 % vs. 46 %, P = 0.018) compared to Group II. At baseline, estimated GFR (91.7 ± 20.3 vs. 98.4 ± 22.9 ml/min/1.73m^2^, P = 0.079) and ACR [47.3 (11.5–136.1) vs. 14.6 (6.0–41.2) μg/mg, P = 0.004] differed between the groups without and with significance, respectively. However, serum creatinine levels [0.8 (0.6–0.9) vs. 0.8 (0.7–0.9) mg/dL, P = 0.864] were the same in the two groups. Although mean HbA_1c_ levels (9.0 ± 1.4 vs. 8.7 ± 1.7 %, P = 0.088) were not significantly different, the mean GA levels [29.3 (22.8–34.7) vs. 24.0 (21.0–27.5) %, P = 0.004] were significantly higher in Group I.

**Table 1:** Demographic and laboratory characteristics of patients

	**All**	**Group I**	**Group II**	
		**DN progression**	**DN non**-**progression**	**P**
	**(n = 154)**	**(n = 30)**	**(n = 124)**	
**Demographics**				
Age (years)	46 ± 15	50 ± 15	45 ± 16	0.135
Male Sex, n (%)	69 (45)	11 (37)	58 (47)	0.414
BMI (kg/m^2^)	22.6 ± 3.3	23.2 ± 3.5	22.4 ± 3.3	0.256
Obesity, n (%)	30 (19)	8 (27)	22 (18)	0.306
Duration of diabetes (years)	10.0 (3.0–15.0)	12.0 (10.0–18.0)	8.0 (3.0–15.0)	0.004
Retinopathy, n (%)	78 (51)	21 (70)	57 (46)	0.018
Hypertension, n (%)	31 (20)	9 (30)	22 (18)	0.202
ARB or ACEI use, n (%)	49 (32)	11 (37)	38 (31)	0.663
Statin use, n (%)	54 (35)	15 (50)	39 (31)	0.056
**Glycemic indices**				
mean GA (%)	24.4 (21.5–29.0)	29.3 (22.8–34.7)	24.0 (21.0–27.5)	0.004
mean HbA_1c_ (%)	8.7 ± 1.6	9.0 ± 1.4	8.7 ± 1.7	0.088
**Renal function indices**				
Baseline ACR (μg/mg)	17.8 (7.2–71.1)	47.3 (11.5–136.1)	14.6 (6.0–41.2)	0.004
Follow-up ACR (μg/mg)	13.8 (7.5–77.9)	134.7 (51.7–766.2)	10.6 (6.8–29.2)	<0.001
Baseline eGFR (mL/min/1.73 m^2^)	97.1 ± 22.5	91.7 ± 20.3	98.4 ± 22.9	0.079
Follow-up eGFR (mL/min/1.73 m^2^)	95.1 ± 26.0	73.9 ± 28.7	100.4 ± 22.4	<0.001
Baseline CKD status				
Stage 1	98 (64)	17 (57)	81 (65)	0.565
Stage 2	48 (31)	12 (40)	36 (29)	
Stage 3 and 4	8 (5)	1 (3)	7 (6)	
**Biochemistry profiles**				
Albumin (g/dL)	4.2 ± 0.4	4.1 ± 0.5	4.3 ± 0.4	0.106
Total cholesterol (mg/dL)	162.0 (145.0–194.0)	171.5 (149.0–195.8)	162.0 (144.0–193.5)	0.423
Triglyceride (mg/dL)	79.0 (60.0–113.5)	90.5 (71.8–118.5)	77.0 (57.0–114.0)	0.109
HDL-cholesterol (mg/dL)	57.0 ± 15.9	55.0 ± 13.7	57.5 ± 16.4	0.669
LDL-cholesterol (mg/dL)	95.5 ± 34.9	97.6 ± 31.3	95.0 ± 35.8	0.495

Continuous variables were described as median (quartiles) or mean ± SD. N (%) for categorical variables

*BMI* body mass index; *ARB* angiotensin II receptor blocker, *ACEI* angiotensin-converting enzyme inhibitor, *ACR* albumin-creatinine ratio, *eGFR* estimated glomerular filtration rate, *CKD* chronic kidney disease

### Independent Association of Diabetic Nephropathy Progression with Glycemic Indices and Risk Variables for Chronic Kidney Disease

**Fig. 1:**
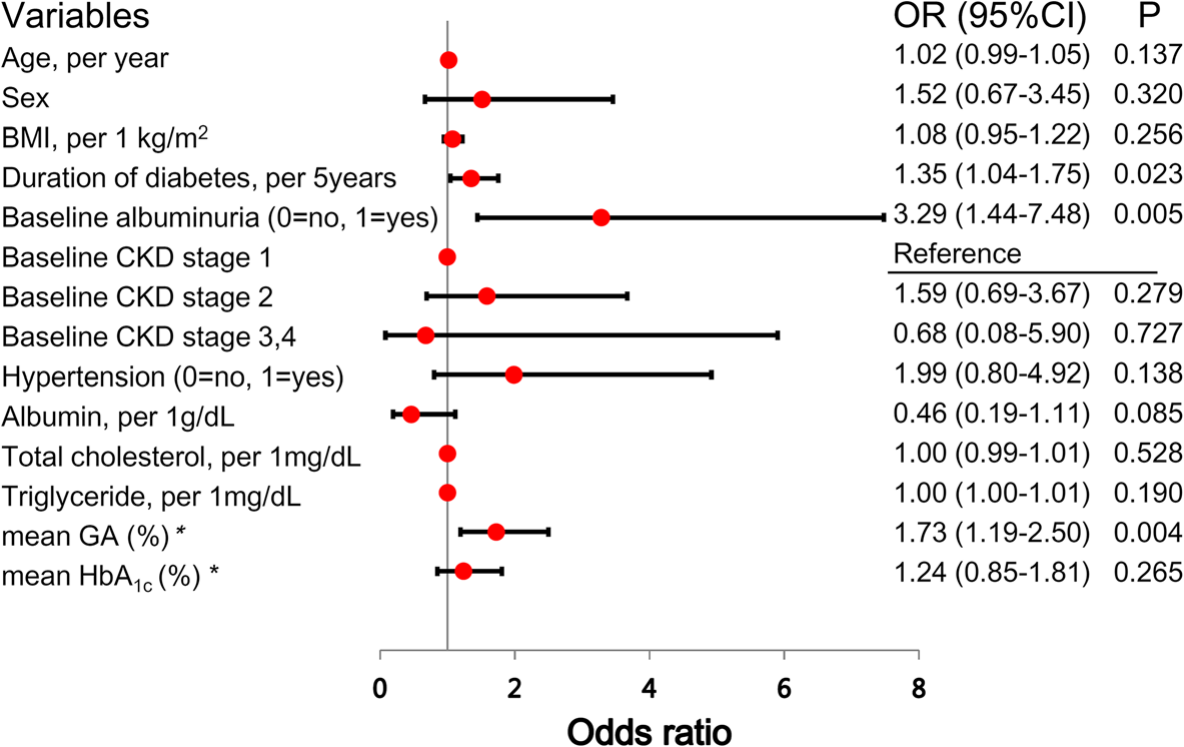
Univariate logistic regression analyses of associations between clinical and biochemical variables and the progression of diabetic nephropathy

In univariate logistic regression analyses, DN progression was used as a dependent factor, and the variables for mean glycemic indices and risk factors for CKD were entered. The risk of progression of DN was 35 % higher in subjects with a prolonged duration of diabetes and as duration increased per 5years (OR = 1.35, 95 % CI 1.04–1.75, P = 0.023). Higher baseline levels of albuminuria (OR = 3.29, 95 % CI 1.44–7.48, P = 0.005) and mean GA levels (OR = 1.73, 95 % CI 1.19–2.50, P = 0.004) were also significantly associated with DN progression. However, mean HbA_1c_ levels (OR = 1.24, 95 % CI 0.85–1.81, P = 0.265) were not significantly related to the progression of DN. Moreover, hypertension, CKD stages, and lipid profiles at baseline were not significantly related to DN progression in this study (Fig. 1).

### Glycated albumin predicted the Progression of Diabetic Nephropathy

Based on the results in Fig. 1, multiple logistic regression analyses were performed to predict the progression of DN (Table 2). For multiple logistic regression analyses, we used four statistical models with different glycemic variables: GA levels and HbA_1c_. In model 1, we entered age, duration per 5years, baseline albuminuria, obesity (BMI ≥ 25kg/m^2^), hypertension, baseline CKD stage, and mean GA level. The duration of diabetes (OR = 1.44, 95 % CI 1.01–2.07, P = 0.047), baseline albuminuria (OR = 2.63, 95 % CI 1.03–6.73, P = 0.043), and mean GA level (OR = 2.02, 95 % CI 1.28–3.17, P = 0.002) were significantly associated with the progression of DN. In model 2 with mean HbA_1c_, DN progression did not independently correlate with mean HbA_1c_ (OR = 1.21, 95 % CI 0.79–1.85, P = 0.390). To investigate the effects of both serum GA levels and HbA_1c_ on DN progression, we entered mean GA and mean HbA_1c_ as independent glycemic variables in model 3. Mean GA levels (OR = 2.03, 95 % CI 1.27–3.26, P = 0.003) remained its significance in predicting the progression of DN, whereas mean HbA_1c_ (OR = 0.98, 95 % CI 0.62–1.54, P = 0.918) did not. In terms of retinopathy, duration of diabetes and albuminuria were significant determinants for predicting retinopathy at baseline (data not shown). However, neither mean levels of GA nor HbA_1c_ was associated with the presence of retinopathy at baseline (data not shown).

**Table 2:** Multiple logistic regression models for associations between clinical and biochemical variables and the progression of diabetic nephropathy in a total of 154 subjects with T1D

	**Model 1**			**Model 2**			**Model 3**		
	**(R** ^**2**^ **= 0.28)**			**(R** ^**2**^ **= 0.20)**			**(R** ^**2**^ **= 0.28)**		
**Variables**	**OR**	**95 % CI**	**P**	**OR**	**95 % CI**	**P**	**OR**	**95 % CI**	**P**
Age, per year	1.03	0.99–1.07	0.132	1.03	0.99–1.06	0.188	1.03	0.99–1.07	0.136
Obesity (0 = no, 1 = yes)	1.92	0.64–5.78	0.243	1.95	0.69–5.55	0.210	1.94	0.64–5.81	0.242
Duration of diabetes, per 5years	1.44	1.01–2.07	0.047	1.28	0.92–1.77	0.145	1.44	1.01–2.07	0.047
Hypertension (0 = no, 1 = yes)	2.32	0.77–6.99	0.134	2.09	0.79–5.83	0.160	2.32	0.77–6.98	0.136
Albuminuria at baseline (0 = no, 1 = yes)	2.63	1.03–6.73	0.043	2.86	1.16–7.07	0.023	2.64	1.03–6.74	0.043
Statin use (0 = no, 1 = yes)	1.89	0.74–4.88	0.186	1.49	0.59–3.75	0.403	1.91	0.73–5.01	0.188
CKD stage at baseline									
Stage1	Reference			Reference			Reference		
Stage2	0.55	0.16–1.86	0.338	0.71	0.23–2.24	0.559	0.55	0.16–1.86	0.337
Stage3 and 4	0.09	0.01–1.30	0.078	0.12	0.01–1.54	0.103	0.09	0.01–1.31	0.078
mean GA (%)*	2.02	1.28–3.17	0.002				2.03	1.27–3.26	0.003
mean HbA_1c_ (%)*				1.21	0.79–1.85	0.390	0.98	0.62–1.54	0.918

*CKD* chronic kidney disease

*z-standardization of glycemic indices

**Fig. 2:**
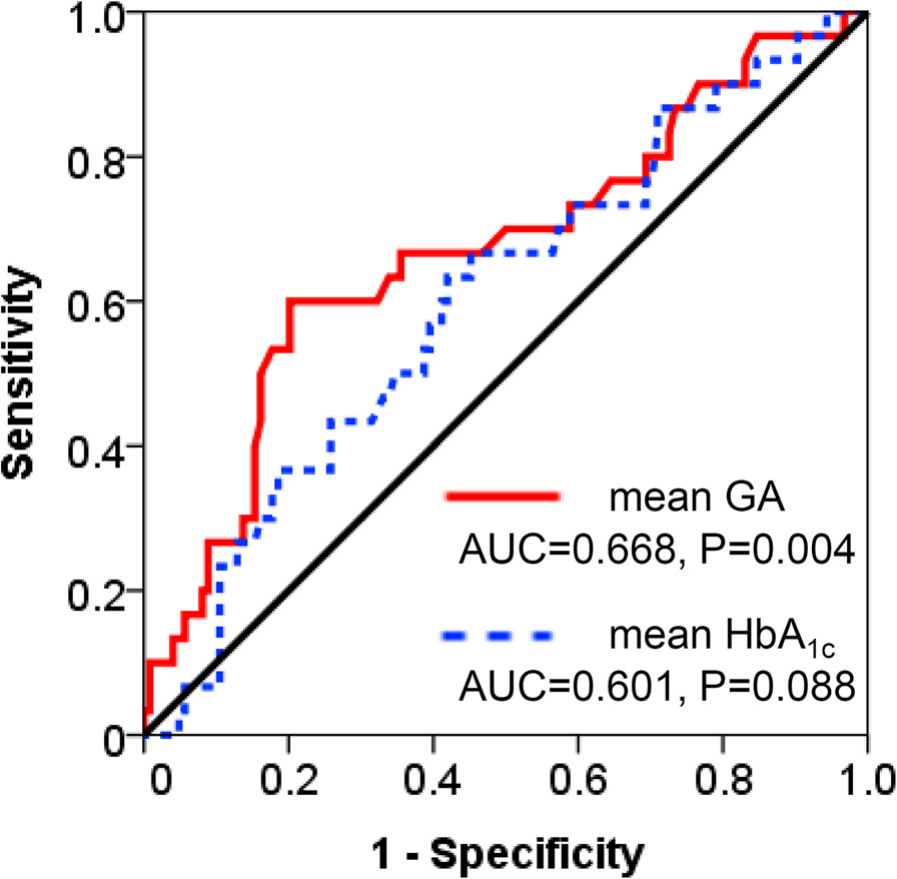
Receiver operating characteristic curve of mean HbA_1c_ and GA levels for predicting diabetic nephropathy progression

Using ROC curve analyses, we calculated the AUC of glycemic indices (mean GA and mean HbA_1c_) for predicting DN progression (Fig. 2). Mean GA levels showed a larger AUC (AUC = 0.668, 95 % CI 0.55–0.78, P = 0.004) compared to mean HbA_1c_ (AUC = 0.601, 95 % CI 0.49–0.71, P = 0.088). The AUC of mean GA levels was statistically significant.

### Association of Carotid artery atherosclerosis with Glycemic Indices and Cardiovascular Risk Factors

Of the 154 patients with T1D, 54 subjects had repeated carotid IMT data with a mean 2.9 year follow-up. Subjects were classified into the active CAA group (n = 22) and the inactive CAA group (n = 32). Age [56 (50–70) vs. 41 (35–48) years, P < 0.001] and triglyceride levels [103.5 (63.8–189.8) vs. 72.5 (55.3–89.8) mg/dL, P = 0.004] were significantly higher in the active CAA group. Compared with the inactive CAA group, the active CAA group showed significantly higher ACR levels at both baseline [28.7 (9.8–136.1) vs. 7.7 (4.1–21.5) μg/mg] and follow-up [32.0 (10.0–108.8) vs. 9.1 (2.9–24.1) μg/mg], as well as lower estimated GFR at baseline [87.6 (67.4–100.2) vs. 103.1 (82.9–115.3) mL/min/1.73m^2^, P = 0.008] and follow-up [86.9 (69.8–96.8) vs. 102.7 (83.6–112.8) mL/min/1.73m^2^, P = 0.030] (Additional file 1: Table S1).

We used the multiple logistic regression model (model A) for carotid artery plaques and the multiple linear regression model (model B) for follow-up mean carotid IMT values to determine the clinically independent variables that were associated with carotid artery plaques. In both models, only age (model A: OR 1.20, 95 % CI 1.05–1.36, P = 0.007, and model B: standardized β = 0.58, P < 0.001) was significantly associated with CAA. In these models, glycemic indices, including mean GA levels and mean HbA_1c_, and renal function related variables were not significantly related to CAA (Table 3).

**Table 3:** Associations of the cardiovascular risk factors with carotid artery atherosclerosis in a subgroup of 54 subjects with T1D

	**Model A**			**Model B**	
	**Presence of plaque at follow up**			**Follow up IMT mean value**	
**Variables**	**OR**	**95 % CI**	**P**	**STD β**	**P**
Age, per year	1.20	1.05–1.36	0.007	0.58	<0.001
Sex (0 = female, 1 = male)	0.92	0.13–6.47	0.933	0.01	0.915
Obesity (0 = no, 1 = yes)	6.90	0.73–65.64	0.093	0.13	0.251
Duration of diabetes, per 5years	1.39	0.74–2.61	0.301	−0.02	0.886
Hypertension (0 = no, 1 = yes)	5.20	0.60–44.98	0.135	0.03	0.801
Albuminuria at baseline (0 = no, 1 = yes)	4.61	0.60–35.57	0.143	0.02	0.876
Baseline eGFR , per 1mL/min/1.73 m^2^	1.05	0.97–1.12	0.219	−0.09	0.590
LDL-cholesterol, per 1mg/dL	0.98	0.94–1.02	0.252	0.15	0.268
mean GA (%)*	0.43	0.09–2.00	0.280	−0.16	0.124
mean HbA_1c_ (%)*	0.53	0.21–1.36	0.187	0.16	0.168

Model A: multiple logistic regression analysis, Model B: multiple linear regression analysis

*eGFR* estimated glomerular filtration rate, *IMT* intima-media thickness, *STD* standardized

*z-standardization of glycemic indices

## Discussion

This study focused on the association between glycemic indices and the progression of diabetic micro- and macrovascular complications in Korean subjects with T1D. The availability of longitudinal measurements for intermediate- and long-term glycemic indices, as well as for diabetic micro- and macrovascular complications, enabled us to investigate the associations between glycemic indices (GA levels and HbA_1c_) and the progression of DN and CAA in T1D. The longitudinal nature also conferred the availability of mean glycemic indices as time-dependent covariates.

Conventionally, DN is defined as a progressive kidney disease characterized by persistent albuminuria, increased blood pressure, and a continuous decline in GFR. It is a common cause of ESRD and has a high risk of cardiovascular morbidity and mortality [17, 18]. Although persistent albuminuria is a strong predictor for DN progression, impaired GFR is possible in the absence of progression to proteinuria in T1D subjects with microalbuminuria [19]. Furthermore, the progression of albuminuria might be critical but not essential in CKD progression [20]. Based on these findings, in this study, we defined the progressed DN group as either deteriorated estimated GFR based CKD stages or the advent of microalbuminuria or progression of albuminuria stage [21, 22]. In addition, based on our previous cross-sectional study [23], we classified the available subjects into active and inactive CAA progression groups.

Considering the role of GA as a short-term (3-week) glycemic parameter, GA can reflect glucose fluctuation and postprandial glucose more sensitively than HbA_1c_ [24, 25]. To investigate the association between the glycemic indices and the progression of diabetic micro- and macrovascular complications, we used the following analyses. To validate the reliability of glycemic indices in this study, we assessed correlations between HbA_1c_ and GA levels at baseline. As expected, HbA_1c_ and GA levels were highly correlated at baseline (r = 0.631, P < 0.001). Next, we examined the association between glycemic indices and progression of DN and CAA. Recent studies reported that serum GA is closely associated with, or predicts, DN and diabetic retinopathy in patients with both T2D [26, 27] and T1D [11]. However, the clinical implications of GA on the presence or progression of CAA was not noted T1D patients [11] but was observed in T2D patients [7, 15]. Furthermore, GA but not HbA_1c_ was associated with coronary heart disease in the Chinese population with exclusion of T1D [28]. In a case-cohort subpopulation of the DCCT [11], HbA_1c_ and GA levels had similar associations with retinopathy and nephropathy, but only HbA_1c_ was significantly associated with cardiovascular disease. Selvin et al., also demonstrated the significant association of GA with incident CKD in subjects with both type 1 and 2 diabetes even after adjustment with HbA_1c_ [29], indicating that GA can work well for predicting microvascular complications [30]. This is supported by another study showing increased levels of GA in subjects with retinopathy [31]. This study has three main findings: first, diabetes duration fundamentally affected the progression of DN; second, albuminuria levels, not baseline CKD stage, were significantly related to DN progression; third, mean GA, but not mean HbA_1c_, were significantly associated with progression of DN. Regarding CAA as a macrovascular complication, HbA_1c_ and GA levels were not related to the progression of carotid IMT. Consistent with previous studies [21, 22], age was the only factor that was significantly associated with CAA in this study.

Although there was no association between HbA_1c_ and DN, this does not imply that poor glycemic control does not affect diabetic microvascular complications. Considering glycemic control and the clinical outcomes in T1D, the DCCT/EDIC study clearly confirmed that patients with T1D in the intensive treatment group with lower HbA_1c_ levels had reduced risks of microalbuminuria and declines in GFR compared to patients in the conventional treatment group [2, 3, 5]. DN progression was assessed using multiple logistic regression models, and the association with mean HbA_1c_ was further attenuated once mean GA levels were also entered; however, mean GA levels might be augmented when HbA_1c_ was considered [11]. These findings suggest that GA measurements might be helpful in predicting DN in subjects with T1D.

With respect to the effects of GA on the progression of DN and CAA in T1D, we hypothesize that different complications might be affected by hyperglycemia. By pathophysiological, T1D is a disease caused by absolute insulin deficiency; however, the primarily underlying pathophysiologic mechanism of T2D is hyperinsulinemic insulin resistance [32]. Clinically, DN is more prone to glucotoxicity than insulin resistance. In addition, metabolic syndrome, primarily caused by insulin resistance, is a known risk factor for DN [33]. However, in this study, BMI and triglyceride levels were not related to DN progression. The low BMI of patients (Group I; mean BMI = 23.2 kg/m^2^ and Group II; mean BMI = 22.4 kg/m^2^) in our study population might account for the insignificant association between DN progression and variables for metabolic syndrome. Therefore, the statistical association of GA with the progression of DN but not with CAA is not surprising. We postulate that the clinical relevance of GA levels on the development of CAA in T1D might be in contrast to the presence of insulin resistance observed in T2D. Based on these findings, we postulate that glucotoxicity might be important for the progression of DN, but insulin resistance might play a major role in the development of CAA regardless of diabetes type.

With respect to results on the relationship of glycemic indices with the progression of DN and CAA, our findings generally coincide with the data from previous studies; however, some conflicts remain. Possible explanations for our differing results are the differences in the baseline characteristics of patients and the longitudinal observation study design. The enrolled subjects had primarily adult-onset T1D (average age 46 years with 10 years of diabetes duration) with glycemia that was not well controlled (mean HbA_1c_, 8.7 %). They were followed up for a median of 2.8 years to assess the progression of DN and CAA. Although mean creatinine (0.8 mg/dL) and estimated GFR (97.1 ml/min/1.73m^2^) levels were in the apparently normal range, this study included 8 subjects (5 %) with CKD (except for ESRD on dialysis). Because HbA_1c_ does not reflect blood glucose levels accurately in subjects with advanced renal disease [10], GA levels might more accurately reflect the glycemic status in these subjects. These differences in baseline characteristics and study design might account for the different results from previous studies.

This study had some limitations. First, the follow-up period was relatively short (2.8 years) and small number of T1D subjects which led to have not enough estimated power for this study (68 %). Second, we did not analyze the influence of smoking habits or medication status that could affect GA levels. Third, we did not measure blood pressure repeatedly; therefore, the effect of continuous blood pressure was not included. Despite these limitations, our study population consists primarily of patients with adult-onset T1D, which is not a common study population in the T1D research field. The tools for assessing glycemic indices (HbA_1c_ and GA) and diabetes complications (ACR and carotid IMT) are well documented. Moreover, this is the first longitudinal observation study for Asian subjects with T1D that examines the clinical relationship between glycemic indices and the outcomes of diabetic complications.

In summary, we investigated the association of various glycemic indices and the progression of DN and CAA in Korean subjects with T1D. Mean GA levels, rather than mean HbA_1c_, are more closely associated with the progression of DN. However, all glycemic indices were not associated with the progression of CAA. In conclusion, we suggest that GA levels, rather than HbA_1c_, are associated with DN progression and confer clinical relevance in the management of T1D. Well-designed prospective studies enrolling larger populations are warranted.

## Additional file

Additional file 1: Table S1. Baseline characteristics of patients in which carotid intima-media-thickness was measured.
