# Supplementary material for: Glycated albumin and the risk of micro- and macrovascular complications in subjects with Type 1 Diabetes
Source: Cardiovasc Diabetol. 2015 May 15;14:53. doi: 10.1186/s12933-015-0219-y (PMC4438622; doi:10.1186/s12933-015-0219-y)
Supplement: Additional file 1: Table S1. — Baseline characteristics of patients in which carotid intima-media-thickness was measured. [file 12933_2015_219_MOESM1_ESM.docx]

**Supplementary Table1** Baseline characteristics of patients in which carotid intima-media-thickness was measured

|  | **All** | |  | **Carotid artery atherosclerosis group** | |  | **Non-carotid artery atherosclerosis group** | | **P** | |
| --- | --- | --- | --- | --- | --- | --- | --- | --- | --- | --- |
| **Variables** | **(n=54)** | |  | **(n=22)** | |  | **(n=32)** | |  |  |
| **Demographics** |  |  |  |  |  |  |  |  |  |  |
| Age (years) | 47 | (39-56) |  | 56 | (50-70) |  | 41 | (35-47.8) |  | <0.001 |
| Male Sex, n (%) | 24 | (44) |  | 10 | (46) |  | 14 | (44) |  | 0.901 |
| BMI (kg/m^2^) | 23.0 | (21.6-25.8) |  | 23.8 | (22.2-25.6) |  | 22.9 | (20.7-26.0) |  | 0.241 |
| Duration of diabetes (years) | 8 | (2-15) |  | 12 | (4.5-19.0) |  | 6 | (1-13) |  | 0.083 |
| Retinopathy, n (%) | 26 | (48) |  | 13 | (59) |  | 13 | (41) |  | 0.108 |
| Hypertension, n (%) | 16 | (30) |  | 9 | (41) |  | 7 | (22) |  | 0.225 |
| ARB or ACEI use, n (%) | 18 | (33) |  | 7 | (32) |  | 11 | (34) |  | 0.042 |
| Statin use, n (%) | 37 | (69) |  | 13 | (59) |  | 24 | (75) |  | 0.216 |
|  |  |  |  |  |  |  |  |  |  |  |
| **Glycemic indices** |  |  |  |  |  |  |  |  |  |  |
| mean GA (%) | 25.4 | (23.0-29.6) |  | 25.3 | (22.8-30.0) |  | 25.4 | (23.1-28.8) |  | 0.673 |
| mean HbA_1c_ (%) | 8.7 | (7.9-9.4) |  | 8.7 | (8.0-9.6) |  | 8.8 | (7.9-9.2) |  | 0.751 |
|  |  |  |  |  |  |  |  |  |  |  |
| **Carotid IMT** |  |  |  |  |  |  |  |  |  |  |
| Baseline mean IMT value (mm) | 0.62 | (0.5-0.7) |  | 0.71 | (0.7-0.8) |  | 0.55 | (0.5-0.6) |  | <0.001 |
| Follow-up mean IMT value (mm) | 0.61 | (0.5-0.7) |  | 0.74 | (0.6-0.8) |  | 0.52 | (0.5-0.6) |  | <0.001 |
| Baseline plaque, n (%) | 18 | (33) |  | 18 | (82) |  | 0 | (0) |  | <0.001 |
| Follow-up plaque, n (%) | 22 | (41) |  | 22 | (100) |  | 0 | (0) |  | <0.001 |
|  |  |  |  |  |  |  |  |  |  |  |
| **Renal function indices** |  |  |  |  |  |  |  |  |  |  |
| Baseline ACR (μg/mg) | 12.9 | (5.3-53.6) |  | 28.7 | (9.8-136.1) |  | 7.7 | (4.1-21.5) |  | 0.004 |
| Follow-up ACR (μg/mg) | 10.9 | (6.4-77.5) |  | 32.0 | (10.0-108.8) |  | 9.1 | (2.9-24.1) |  | 0.015 |
| Baseline eGFR (mL/min/1.73 m^2^) | 96.9 | (39.4-131.3) |  | 87.6 | (67.4-100.2) |  | 103.1 | (82.9-115.3) |  | 0.008 |
| Follow-up eGFR(mL/min/1.73m^2^) | 93.8 | (78.0-110.0) |  | 86.9 | (69.8-96.8) |  | 102.7 | (83.6-112.8) |  | 0.030 |
|  |  |  |  |  |  |  |  |  |  |  |
| **Biochemistry profiles** |  |  |  |  |  |  |  |  |  |  |
| Creatinine (mg/dL) | 0.8 | (0.7-1.0) |  | 0.8 | (0.7-1.1) |  | 0.8 | (0.7-0.9) |  | 0.428 |
| Albumin (g/dL) | 4.3 | (4.1-4.5) |  | 4.4 | (4.1-4.5) |  | 4.3 | (4.0-4.6) |  | 0.335 |
| Total cholesterol (mg/dL) | 162.5 | (148.0-195.3) |  | 158.0 | (144.0-202.3) |  | 171.0 | (151.0-193.0) |  | 0.826 |
| Triglyceride (mg/dL) | 85.0 | (60.8-113.3) |  | 103.5 | (63.8-189.8) |  | 72.5 | (55.3-89.8) |  | 0.004 |
| HDL-cholesterol (mg/dL) | 57.5 | (47.5-68.3) |  | 49.0 | (41.0-61.5) |  | 62.0 | (54.3-70.0) |  | 0.005 |
| LDL-cholesterol (mg/dL) | 87.4 | (70.7-103.9) |  | 85.0 | (67.0-108.2) |  | 90.4 | (75.9-102.3) |  | 0.805 |

Continuous variables were described as median (quartiles) or mean ± SD. N (%) for categorical variables. BMI, body mass index; ARB; angiotensin II receptor blocker; ACEI, angiotensin-converting enzyme inhibitor; IMT, intima-media thickness ACR, albumin-creatinine ratio; eGFR, estimated glomerular filtration rate
